# Bis(μ_2_-η^2^:η^2^-2,4,6-trimethyl­benzonitrile)­bis­[(*N*-isopropyl-3,5-dimethyl­anilido)molybdenum(III)](*Mo*—*Mo*)

**DOI:** 10.1107/S1600536811044680

**Published:** 2011-11-02

**Authors:** Yurii S. Moroz, Anthony F. Cozzolino, Elena V. Rybak-Akimova, Christopher C. Cummins

**Affiliations:** a Department of Chemistry, Tufts University, 62 Talbot Avenue, Medford, MA 02155, USA; b Department of Chemistry, Massachusetts Institute of Technology, 77 Massachusetts Avenue, Cambridge, MA 02139, USA

## Abstract

The title compound, [Mo_2_(C_11_H_16_N)_4_(C_10_H_11_N)_2_], is a dinuclear molybdenum complex with a formal metal–metal bond [Mo⋯Mo separation = 2.5946 (8) Å], four anilide-type ligands and two bridging mesityl nitrile groups. There are two inversion symmetric mol­ecules in the unit cell (an inversion center is localized at the mid-point of the Mo—Mo bond), each with approximate non-crystallographic *C*_2*h*_ symmetry. The mol­ecules contain disordered isopropyl and 3,5-C_6_H_3_Me_2_ groups on different anilido ligands; the major component having an occupancy of 0.683 (7). The complex was obtained in low yield as the product from the reaction between the bridging pyrazine adduct of molybdenum *tris­*-anilide ([μ_2_-(C_4_H_4_N_2_){Mo(C_11_H_16_N)_3_}_2_]) and mesityl nitrile with a loss of one anilido ligand.

## Related literature

For the synthesis of molybdenum(III) *tris­*-anilide nitrides and structures of similar complexes, see: Johnson *et al.* (1997 ▶); Tsai *et al.* (1999 ▶). For reactions of three-coordinate Mo(III) complexes with dinitro­gen, organic nitriles and isocyanides, including a base-catalysed dinitro­gen cleavage, see: Tsai *et al.* (2003 ▶); Curley *et al.* (2008 ▶); Germain *et al.* (2009 ▶). For the structural parameters of mesityl nitrile and its complexes, see: Britton (1979 ▶); Figueroa & Cummins (2003 ▶). For the structural parameters of molybdenum complexes with μ_2_-η^2^-η^2^ bridging benzonitrile, see: Li *et al.* (2008 ▶).
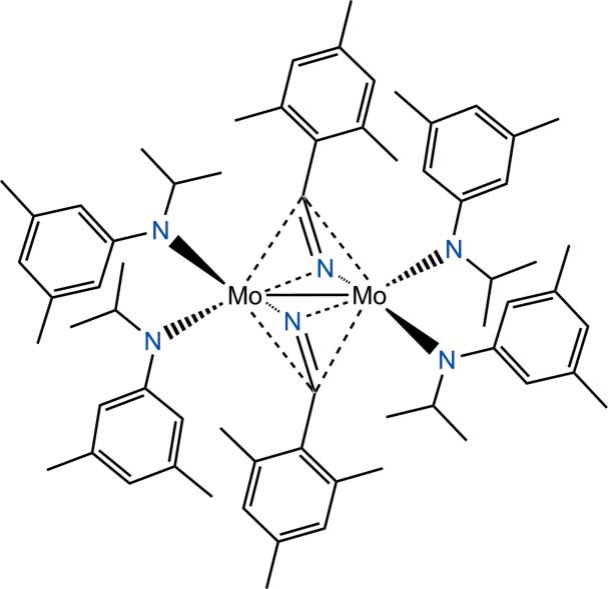


## Experimental

### 

#### Crystal data

[Mo_2_(C_11_H_16_N)_4_(C_10_H_11_N)_2_]
*M* *_r_* = 1131.27
Monoclinic, 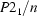

*a* = 13.262 (2) Å
*b* = 17.090 (3) Å
*c* = 13.306 (2) Å
β = 109.387 (2)°
*V* = 2844.7 (8) Å^3^
*Z* = 2
Mo *K*α radiation
μ = 0.49 mm^−1^
*T* = 100 K
0.15 × 0.1 × 0.07 mm

#### Data collection

Bruker SMART APEX CCD diffractometer
Absorption correction: multi-scan (*SADABS*; Sheldrick, 2009 ▶) *T*_min_ = 0.459, *T*_max_ = 0.746
55443 measured reflections
7345 independent reflections
4190 reflections with *I* > 2σ(*I*)
*R*_int_ = 0.119

#### Refinement

*R*[*F* ^2^ > 2σ(*F* ^2^)] = 0.067
*wR*(*F* ^2^) = 0.172
*S* = 1.08
7345 reflections
375 parameters
63 restraints
H-atom parameters constrained
Δρ_max_ = 1.54 e Å^−3^
Δρ_min_ = −1.51 e Å^−3^

### 

Data collection: *APEX2* (Bruker, 2009 ▶); cell refinement: *SAINT* (Bruker, 2009 ▶); data reduction: *SAINT*; program(s) used to solve structure: *SHELXS97* (Sheldrick, 2008 ▶); program(s) used to refine structure: *SHELXL97* (Sheldrick, 2008 ▶); molecular graphics: *ORTEP-3 for Windows* (Farrugia, 1997 ▶); software used to prepare material for publication: *WinGX* (Farrugia, 1999 ▶).

## Supplementary Material

Crystal structure: contains datablock(s) I, global. DOI: 10.1107/S1600536811044680/zl2410sup1.cif

Structure factors: contains datablock(s) I. DOI: 10.1107/S1600536811044680/zl2410Isup2.hkl

Additional supplementary materials:  crystallographic information; 3D view; checkCIF report

## Figures and Tables

**Fig. 1.**
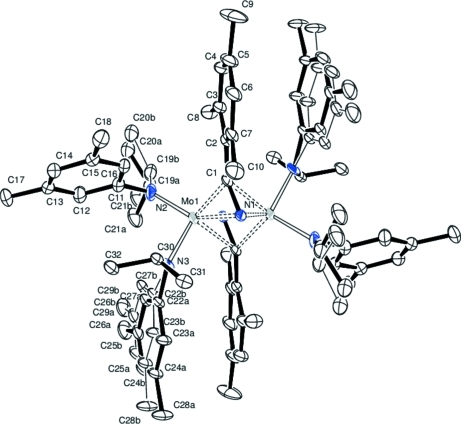
An ORTEP view of the title compound, with displacement ellipsoids shown at the 40% probability level (Hydrogen atoms are omitted for clarity). The minor component of the disordered Ar and i-Pr groups is shown with line bonds. Symmetry transformations used to generate equivalent atoms: -x+2, -y+2, -z+1.

**Fig. 2.**
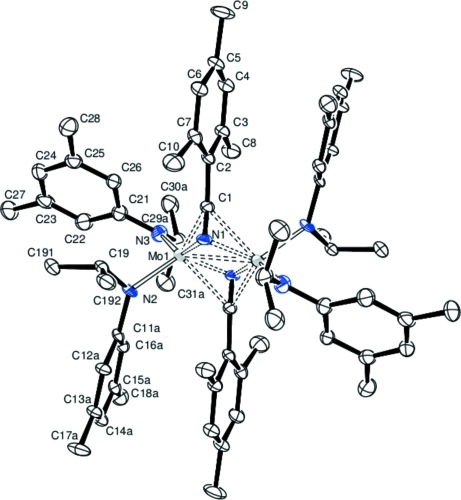
An ORTEP view of the title compound, with displacement ellipsoids shown at the 40% probability level (Hydrogen atoms and the minor component of the disordered Ar and i-Pr groups are omitted for clarity). Symmetry transformations used to generate equivalent atoms: -x+2, -y+2, -z+1.

**Fig. 3.**
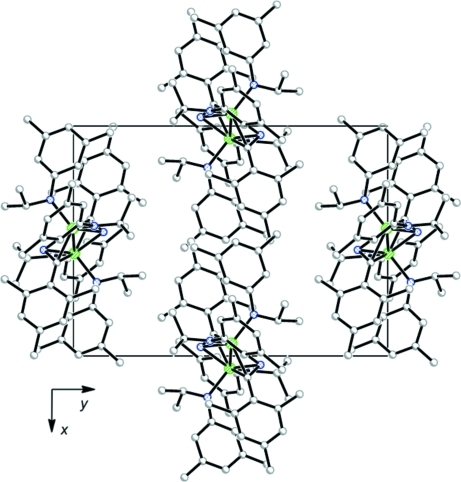
A packing diagram for the title compound viewed along the [001] axis (H atoms and the minor component of the disordered Ar and i-Pr groups are omitted for clarity).

## supplementary crystallographic information

### Comment

For more than fifteen years, low-coordinate molybdenum(III) *tris*-anilide complexes, [Mo(N{*R*_1_}Ar)_3_] and [HMo(η_2_-Me_2_CNAr)(N{*R*_2_}Ar)_2_] (where *R*_1_ = *t*-Bu; *R*_2_ = *i*-Pr or CH(CD_3_)_2_; Ar = 3,5-C_6_H_3_Me_2_), have attracted the attention of inorganic chemists and crystallographers due to their unusual coordination geometries and their remarkable ability to activate small molecules, including triply-bonded dinitrogen (Tsai *et al.*, 1999; Curley *et al.*, 2008; Germain *et al.*, 2009). It was previously shown that N_2_ cleavage with the sterically bulky [Mo(N{*t*-Bu}Ar)_3_] affords a terminal nitride, ([(N)Mo(N{*t*-Bu}Ar)_3_]), while the less bulky [HMo(η_2_-(CD_3_)_2_CNAr)(N{CH(CD_3_)_2_}Ar)_2_] yields a µ_2_-N bridged dinuclear complex ([µ_2_-(N){Mo(N{CH(CD_3_)_2_}Ar)_3_}_2_]). Furthermore, the rate of N_2_ uptake increases in the presence of bases such as 1-methyl-imidazole, 2,6-dimethylpyrazine or pyridine (Tsai *et al.*, 2003). Thus, the study of molybdenum *tris*-anilide adducts with additional ligands will help in understanding the N_2_ uptake, a critical step in the overall N_2_ cleavage mechanism. Additionally, molecules with element-element triple bonds, such as nitriles, can be viewed as dinitrogen surrogates, and provide structural information on molybdenum interacting with multiply bonded substrates in cases when N_2_ binding affinity is too low, and N_2_ complexes cannot be isolated and crystallized.

In this report, we discuss the molecular structure of the dinuclear [µ_2_-η^2^-η^2^-(MesCN)_2_{Mo(N{*i-*Pr}Ar)_2_}_2_] molybdenum compound (Figure 1 and 2) obtained from the reaction between mesityl nitrile and the pyrazine adduct of [HMo(η_2_-Me_2_CNAr)(N{*i*-Pr}Ar)_2_] mixed in 2:1 stoichiometric ratio. The title compound crystallizes in the monoclinic space group *P*2_1_/n and consists of neutral molecules; inter-molecular interactions include a number of van der Waals contacts. The crystal packing diagram reveals that molecules of the title compound form layers in the *xz* plane (Figure 3). The molybdenum centers have distorted tetrahedral geometries; the coordination polyhedron is formed by two N atoms belonging to the anilide residues and two bridging η^2^-CN groups from MesCN molecules. Additionally, short metal-metal separation (2.5946 (8) Å) indicates the presence of a formal single Mo···Mo' bond. The bridging η^2^-η^2^– coordination of MesCN results in an elongation of the C—N bond in the molecule. Its value is typical for a double C—N bond (1.318 (7) Å) and longer than the CN triple bond in free MesCN (1.160 Å, Britton, 1979) and for the case of η^2^-coordination to a single Nb center (1.258 (4) Å, Figueroa *et al.*, 2003) but very close to the value reported for µ_2_-η^2^-η^2^ benzonitrile coordinated to two molybdenum atoms (1.299 (3) Å) (Li, *et al.*, 2008). The Mo—N bond lengths are comparable to the previously reported [Mo(N{*R*}Ar)_3_] complexes (Geometric parameters table) (Johnson *et al.*, 1997), and the other C—C, C—N bond length values in the anilide and mesityl ligands have their typical values.

### Experimental

All synthetic operations were performed in an air-free MBraun drybox under an argon atmosphere. Compound [µ_2_-η^2^-η^2^-(C_10_N_1_H_11_)_2_{Mo(C_11_N_1_H_16_)_2_}_2_] was obtained in two steps. [µ_2_-(pyrazine){Mo(N{*i-*Pr}Ar)_3_}_2_] was synthesized *in situ*: 0.002 g (0.03 mmol) of pyrazine in 2 ml of dry THF was added to a solution of molybdenum complex, [HMo(η_2_-Me_2_CNAr)(N{*i*-Pr}Ar)_2_] (0.03 g, 0.05 mmol) in dry diethyl ether (3 ml). The resulting dark blue mixture was stirred for 20 min, followed by solvent evaporation under reduced pressure. In the second step the crude product was redissolved in diethyl ether and 0.008 g (0.06 mmol) of mesityl nitrile was added. The mixture was stirred for 30 min, then the solvent was evaporated under reduced pressure. The product was redissolved twice in dry *n*-hexane to remove the traces of the THF, then the solvent was again evaporated and the solid was dissolved in dry *n*-pentane (2 ml) and left for crystallization at 238 K (-35 °C) inside the glove box. Dark blue crystals suitable for X-ray analysis formed after one week (Yield (crude): 20%). ^1^H NMR (500 MHz, 298 K, C_6_D_6_): δ -8.94, -1.79, 0.62, 3.06, 8.84, 9.02, 15.96 ppm.

### Refinement

Methyl H atoms were placed in geometrically idealized positions allowing the initial torsion angle to be determined by a difference Fourier analysis [C—H = 0.96 Å and *U*_iso_(H) = 1.5*U*_eq_(C)]. Other H atoms were placed in geometrically idealized positions and included as riding atoms [C—H = 0.93–0.98 Å and *U*_iso_(H) = 1.2*U*_eq_(C)]. Disordered *i*-Pr and disordered aryl groups reside next to each other in the unit cell which causes a correlation in the model of the disorder, the ratios between the two components were refined freely. The geometries of the two PARTs of the disordered *i*-Pr and aryl groups were kept similar using the SAME command of the program Shelxtl (Sheldrick, 2008); additionally, SADI restraints were used to restrain N2—C19A, N2—C19B and N3—C22A, N3—C22B bond distances. This allowed the 1,2- and 1,3-distances of corresponding atoms to be equal within determined standard deviations (0.02 Å for 1,2- and 0.04 for 1,3-distances). Rigid bond restraints for anisotropic displacement parameters of atoms of the disordered *i*-Pr and disordered aryl group were applied using the DELU command (standard deviation is 0.01 Å). In addition, anisotropic displacement parameters of the pairs of overlapping disordered atoms of the major and minor components of the disorder were made equal using the EADP constraint.

### Crystal data

**Table:**

[Mo_2_(C_11_H_16_N)_4_(C_10_H_11_N)_2_]	*F*(000) = 1192
*M**_r_* = 1131.27	*D*_x_ = 1.321 Mg m^−^^3^
Monoclinic, *P*2_1_/*n*	Mo *K*α radiation, λ = 0.71073 Å
Hall symbol: -P 2yn	Cell parameters from 55443 reflections
*a* = 13.262 (2) Å	θ = 1.9–28.7°
*b* = 17.090 (3) Å	µ = 0.49 mm^−^^1^
*c* = 13.306 (2) Å	*T* = 100 K
β = 109.387 (2)°	Plate, dark blue
*V* = 2844.7 (8) Å^3^	0.15 × 0.1 × 0.07 mm
*Z* = 2	

### Data collection

**Table:**

Bruker SMART APEX CCD diffractometer	7345 independent reflections
Radiation source: fine-focus sealed tube	4190 reflections with *I* > 2σ(*I*)
graphite	*R*_int_ = 0.119
φ and ω scans	θ_max_ = 28.7°, θ_min_ = 1.9°
Absorption correction: multi-scan (*SADABS*; Sheldrick, 2009)	*h* = −17→17
*T*_min_ = 0.459, *T*_max_ = 0.746	*k* = −23→23
55443 measured reflections	*l* = −17→17

### Refinement

**Table:**

Refinement on *F*^2^	Secondary atom site location: difference Fourier map
Least-squares matrix: full	Hydrogen site location: inferred from neighbouring sites
*R*[*F*^2^ > 2σ(*F*^2^)] = 0.067	H-atom parameters constrained
*wR*(*F*^2^) = 0.172	*w* = 1/[σ^2^(*F*_o_^2^) + (0.0372*P*)^2^ + 14.5221*P*] where *P* = (*F*_o_^2^ + 2*F*_c_^2^)/3
*S* = 1.08	(Δ/σ)_max_ = 0.003
7345 reflections	Δρ_max_ = 1.54 e Å^−^^3^
375 parameters	Δρ_min_ = −1.51 e Å^−^^3^
63 restraints	Extinction correction: *SHELXL97* (Sheldrick, 2008), Fc^*^=kFc[1+0.001xFc^2^λ^3^/sin(2θ)]^-1/4^
Primary atom site location: structure-invariant direct methods	Extinction coefficient: 0.0011 (2)

### Special details

**Table:**

Geometry. All s.u.'s (except the s.u. in the dihedral angle between two l.s. planes) are estimated using the full covariance matrix. The cell s.u.'s are taken into account individually in the estimation of s.u.'s in distances, angles and torsion angles; correlations between s.u.'s in cell parameters are only used when they are defined by crystal symmetry. An approximate (isotropic) treatment of cell s.u.'s is used for estimating s.u.'s involving l.s. planes.
Refinement. Methyl H atoms were placed in geometrically idealized positions allowing the initial torsion angle to be determined by a difference Fourier analysis [C—H = 0.96 Å and *U*_iso_(H) = 1.5*U*_eq_(C)]. Other H atoms were placed in geometrically idealized positions and included as riding atoms [C—H = 0.93–0.98 Å and *U*_iso_(H) = 1.2*U*_eq_(C)]. Disordered *i*-Pr and disordered aryl group reside next to each other in the unit cell which causes a correlation in the model of the disorder, the ratios between the two components were refined freely. The geometries of the two PARTs of the disordered groups were kept similar using the SAME (it was applied for all atoms in the disordered *i*-Pr and disordered aryl groups) and SADI (it was used to restrain N2–C19A, N2–C19B and N3–C22A, N3–C22B bond distances) restraints of the program Shelxtl (Sheldrick, 2008) which allowed the 1,2- and 1,3- distances of corresponding atoms to be equal within determined standard deviations (0.02 Å for 1,2- and 0.04 for 1,3-distances); rigid bond restraints for anisotropic displacement parameters of atoms of the disordered *i*-Pr and disordered aryl group were applied using the DELU command (standart deviation is 0.01 Å). In addition, anisotropic displacement parameters of the pairs of overlapping disordered atoms of the major and minor components of the disorder were made equal using the EADP constraint.

### Fractional atomic coordinates and isotropic or equivalent isotropic displacement parameters (Å^2^)

**Table:**

	*x*	*y*	*z*	*U*_iso_*/*U*_eq_	Occ. (<1)
Mo1	0.94612 (3)	0.99741 (3)	0.56542 (3)	0.02648 (14)	
N1	1.0387 (3)	0.9050 (3)	0.5088 (3)	0.0293 (11)	
N2	0.9342 (4)	1.0655 (3)	0.6828 (4)	0.0372 (12)	
N3	0.8204 (3)	0.9260 (3)	0.5336 (4)	0.0347 (12)	
C1	1.0935 (4)	0.9466 (3)	0.5926 (4)	0.0265 (12)	
C2	1.1925 (4)	0.9270 (4)	0.6799 (4)	0.0297 (12)	
C3	1.2509 (4)	0.9866 (4)	0.7474 (4)	0.0341 (14)	
C4	1.3495 (4)	0.9667 (4)	0.8245 (5)	0.0423 (16)	
H4	1.3894	1.0060	0.8683	0.051*	
C5	1.3885 (4)	0.8927 (4)	0.8374 (5)	0.0446 (17)	
C6	1.3278 (4)	0.8341 (4)	0.7753 (5)	0.0406 (15)	
H6	1.3528	0.7829	0.7864	0.049*	
C7	1.2296 (4)	0.8488 (4)	0.6962 (4)	0.0319 (13)	
C8	1.2093 (5)	1.0688 (4)	0.7414 (5)	0.0432 (15)	
H8A	1.1801	1.0846	0.6681	0.065*	
H8B	1.2666	1.1034	0.7784	0.065*	
H8C	1.1544	1.0711	0.7736	0.065*	
C9	1.4973 (5)	0.8740 (5)	0.9173 (6)	0.070 (3)	
H9A	1.5517	0.8858	0.8867	0.105*	
H9B	1.5005	0.8195	0.9355	0.105*	
H9C	1.5085	0.9049	0.9803	0.105*	
C10	1.1667 (5)	0.7804 (4)	0.6362 (5)	0.0432 (15)	
H10A	1.1572	0.7858	0.5618	0.065*	
H10B	1.0981	0.7789	0.6459	0.065*	
H10C	1.2047	0.7328	0.6627	0.065*	
C11	0.9237 (5)	1.0201 (4)	0.7667 (4)	0.0363 (15)	
C12	0.8446 (5)	1.0306 (4)	0.8144 (5)	0.0437 (16)	
H12	0.7966	1.0719	0.7923	0.052*	
C13	0.8364 (5)	0.9810 (5)	0.8934 (4)	0.049 (2)	
C14	0.9048 (5)	0.9175 (4)	0.9242 (4)	0.0412 (15)	
H14	0.8969	0.8831	0.9753	0.049*	
C15	0.9845 (4)	0.9039 (4)	0.8803 (4)	0.0370 (14)	
C16	0.9941 (4)	0.9569 (4)	0.8040 (4)	0.0326 (13)	
H16	1.0496	0.9500	0.7766	0.039*	
C17	0.7532 (5)	0.9951 (5)	0.9464 (5)	0.059 (2)	
H17A	0.7841	1.0252	1.0101	0.089*	
H17B	0.7288	0.9458	0.9642	0.089*	
H17C	0.6939	1.0232	0.8985	0.089*	
C18	1.0611 (5)	0.8367 (4)	0.9145 (5)	0.0465 (16)	
H18A	1.1106	0.8467	0.9845	0.070*	
H18B	1.0996	0.8308	0.8653	0.070*	
H18C	1.0221	0.7896	0.9156	0.070*	
C19A	0.9254 (8)	1.1512 (5)	0.6841 (9)	0.038 (2)	0.683 (7)
H19A	0.9672	1.1715	0.6413	0.046*	0.683 (7)
C21A	0.8119 (8)	1.1862 (8)	0.6359 (10)	0.051 (3)	0.683 (7)
H21D	0.7690	1.1704	0.6779	0.076*	0.683 (7)
H21E	0.7799	1.1676	0.5642	0.076*	0.683 (7)
H21F	0.8165	1.2422	0.6356	0.076*	0.683 (7)
C20A	0.9781 (9)	1.1816 (9)	0.7956 (9)	0.046 (2)	0.683 (7)
H20D	0.9347	1.1688	0.8385	0.069*	0.683 (7)
H20E	0.9859	1.2373	0.7936	0.069*	0.683 (7)
H20F	1.0472	1.1579	0.8259	0.069*	0.683 (7)
C19B	0.9603 (19)	1.1499 (10)	0.711 (2)	0.038 (2)	0.317 (7)
H19B	1.0036	1.1651	0.6671	0.046*	0.317 (7)
C20B	1.0263 (19)	1.179 (2)	0.8245 (19)	0.046 (2)	0.317 (7)
H20A	0.9841	1.1746	0.8705	0.069*	0.317 (7)
H20B	1.0456	1.2330	0.8209	0.069*	0.317 (7)
H20C	1.0899	1.1482	0.8522	0.069*	0.317 (7)
C21B	0.8616 (17)	1.2008 (18)	0.670 (2)	0.051 (3)	0.317 (7)
H21A	0.8270	1.1912	0.5952	0.076*	0.317 (7)
H21B	0.8820	1.2549	0.6807	0.076*	0.317 (7)
H21C	0.8134	1.1887	0.7078	0.076*	0.317 (7)
C22A	0.7116 (6)	0.9388 (6)	0.4677 (9)	0.027 (2)	0.683 (7)
C23A	0.6437 (7)	0.8872 (7)	0.3947 (7)	0.033 (2)	0.683 (7)
H23A	0.6672	0.8370	0.3869	0.040*	0.683 (7)
C24A	0.5414 (6)	0.9106 (7)	0.3335 (7)	0.037 (2)	0.683 (7)
C25A	0.5053 (6)	0.9838 (7)	0.3441 (8)	0.040 (2)	0.683 (7)
H25A	0.4364	0.9980	0.3025	0.048*	0.683 (7)
C26A	0.5698 (7)	1.0379 (6)	0.4161 (8)	0.0344 (18)	0.683 (7)
C27A	0.6723 (6)	1.0137 (6)	0.4776 (7)	0.0295 (19)	0.683 (7)
H27A	0.7161	1.0483	0.5269	0.035*	0.683 (7)
C28A	0.4706 (7)	0.8532 (8)	0.2521 (8)	0.057 (3)	0.683 (7)
H28A	0.4025	0.8490	0.2623	0.086*	0.683 (7)
H28B	0.5043	0.8028	0.2615	0.086*	0.683 (7)
H28C	0.4606	0.8719	0.1814	0.086*	0.683 (7)
C29A	0.5277 (8)	1.1158 (7)	0.4255 (8)	0.050 (2)	0.683 (7)
H29A	0.4800	1.1321	0.3573	0.074*	0.683 (7)
H29B	0.5858	1.1523	0.4499	0.074*	0.683 (7)
H29C	0.4898	1.1140	0.4756	0.074*	0.683 (7)
C22B	0.7171 (14)	0.9624 (13)	0.490 (2)	0.027 (2)	0.317 (7)
C23B	0.6383 (14)	0.9158 (13)	0.4197 (18)	0.033 (2)	0.317 (7)
H23B	0.6526	0.8635	0.4106	0.040*	0.317 (7)
C24B	0.5386 (14)	0.9470 (13)	0.3635 (17)	0.037 (2)	0.317 (7)
C25B	0.5201 (15)	1.0238 (12)	0.3792 (18)	0.040 (2)	0.317 (7)
H25B	0.4542	1.0448	0.3397	0.048*	0.317 (7)
C26B	0.5936 (14)	1.0727 (11)	0.4507 (16)	0.0344 (18)	0.317 (7)
C27B	0.6937 (14)	1.0403 (12)	0.5068 (18)	0.0295 (19)	0.317 (7)
H27B	0.7450	1.0707	0.5558	0.035*	0.317 (7)
C28B	0.4540 (16)	0.8961 (16)	0.2848 (18)	0.057 (3)	0.317 (7)
H28D	0.3844	0.9105	0.2860	0.086*	0.317 (7)
H28E	0.4673	0.8421	0.3046	0.086*	0.317 (7)
H28F	0.4570	0.9036	0.2144	0.086*	0.317 (7)
C29B	0.5700 (17)	1.1550 (12)	0.468 (2)	0.050 (2)	0.317 (7)
H29D	0.5978	1.1885	0.4254	0.074*	0.317 (7)
H29E	0.6027	1.1681	0.5418	0.074*	0.317 (7)
H29F	0.4940	1.1621	0.4477	0.074*	0.317 (7)
C30	0.8460 (4)	0.8482 (4)	0.5816 (4)	0.0321 (13)	
H30	0.9189	0.8522	0.6324	0.039*	
C31	0.8491 (5)	0.7815 (4)	0.5064 (5)	0.0420 (15)	
H31A	0.7774	0.7663	0.4658	0.063*	
H31B	0.8862	0.7376	0.5472	0.063*	
H31C	0.8856	0.7985	0.4589	0.063*	
C32	0.7753 (4)	0.8279 (4)	0.6483 (5)	0.0417 (16)	
H32A	0.7785	0.8695	0.6977	0.062*	
H32B	0.8002	0.7803	0.6867	0.062*	
H32C	0.7029	0.8212	0.6022	0.062*	

### Atomic displacement parameters (Å^2^)

**Table:**

	*U*^11^	*U*^22^	*U*^33^	*U*^12^	*U*^13^	*U*^23^
Mo1	0.01402 (18)	0.0447 (3)	0.02243 (19)	0.0035 (2)	0.00838 (13)	0.0022 (3)
N1	0.0137 (17)	0.055 (3)	0.018 (2)	0.006 (2)	0.0045 (16)	0.004 (2)
N2	0.035 (3)	0.054 (3)	0.031 (2)	0.011 (2)	0.022 (2)	0.002 (2)
N3	0.0090 (19)	0.061 (4)	0.034 (2)	0.003 (2)	0.0058 (17)	0.005 (2)
C1	0.011 (2)	0.044 (3)	0.025 (2)	0.007 (2)	0.0070 (18)	0.004 (2)
C2	0.016 (2)	0.051 (4)	0.023 (2)	−0.002 (2)	0.009 (2)	0.001 (2)
C3	0.018 (2)	0.056 (5)	0.027 (2)	0.002 (3)	0.0058 (18)	0.007 (3)
C4	0.022 (3)	0.071 (5)	0.030 (3)	−0.009 (3)	0.003 (2)	0.008 (3)
C5	0.017 (3)	0.069 (5)	0.042 (3)	−0.001 (3)	0.002 (2)	0.019 (3)
C6	0.020 (3)	0.059 (4)	0.042 (3)	0.005 (3)	0.008 (2)	0.016 (3)
C7	0.017 (2)	0.049 (4)	0.033 (3)	0.004 (2)	0.012 (2)	0.004 (3)
C8	0.035 (3)	0.053 (4)	0.036 (3)	−0.006 (3)	0.004 (3)	−0.002 (3)
C9	0.024 (3)	0.084 (6)	0.078 (5)	−0.006 (4)	−0.016 (3)	0.034 (5)
C10	0.033 (3)	0.045 (4)	0.050 (4)	0.015 (3)	0.012 (3)	0.006 (3)
C11	0.032 (3)	0.052 (4)	0.028 (3)	0.002 (3)	0.014 (2)	−0.003 (2)
C12	0.036 (3)	0.066 (5)	0.034 (3)	0.007 (3)	0.018 (3)	0.001 (3)
C13	0.026 (3)	0.098 (6)	0.023 (3)	−0.005 (3)	0.009 (2)	−0.007 (3)
C14	0.033 (3)	0.064 (5)	0.028 (3)	−0.011 (3)	0.013 (2)	0.000 (3)
C15	0.028 (3)	0.058 (4)	0.024 (3)	−0.001 (3)	0.007 (2)	−0.001 (3)
C16	0.028 (3)	0.050 (4)	0.023 (3)	0.002 (3)	0.013 (2)	0.000 (2)
C17	0.032 (3)	0.111 (6)	0.043 (3)	−0.004 (4)	0.024 (3)	0.004 (5)
C18	0.047 (4)	0.058 (4)	0.036 (3)	0.002 (3)	0.016 (3)	0.003 (3)
C19A	0.037 (7)	0.054 (5)	0.027 (6)	0.015 (4)	0.016 (5)	0.010 (4)
C21A	0.036 (6)	0.061 (7)	0.064 (7)	0.017 (6)	0.029 (5)	0.007 (6)
C20A	0.047 (7)	0.057 (5)	0.041 (6)	−0.005 (7)	0.024 (5)	−0.010 (5)
C19B	0.037 (7)	0.054 (5)	0.027 (6)	0.015 (4)	0.016 (5)	0.010 (4)
C20B	0.047 (7)	0.057 (5)	0.041 (6)	−0.005 (7)	0.024 (5)	−0.010 (5)
C21B	0.036 (6)	0.061 (7)	0.064 (7)	0.017 (6)	0.029 (5)	0.007 (6)
C22A	0.012 (2)	0.045 (6)	0.024 (6)	−0.004 (3)	0.007 (2)	0.002 (4)
C23A	0.018 (3)	0.048 (6)	0.026 (5)	−0.008 (3)	−0.002 (3)	0.003 (4)
C24A	0.016 (3)	0.063 (6)	0.026 (5)	−0.008 (4)	−0.001 (3)	0.006 (4)
C25A	0.015 (3)	0.068 (6)	0.035 (5)	0.001 (4)	0.006 (3)	0.016 (4)
C26A	0.019 (4)	0.056 (6)	0.032 (6)	0.006 (4)	0.014 (3)	0.010 (4)
C27A	0.016 (3)	0.047 (6)	0.028 (5)	−0.002 (4)	0.011 (3)	0.001 (4)
C28A	0.027 (4)	0.090 (9)	0.038 (6)	−0.015 (5)	−0.012 (4)	−0.005 (5)
C29A	0.035 (5)	0.068 (6)	0.052 (6)	0.020 (4)	0.023 (5)	0.010 (4)
C22B	0.012 (2)	0.045 (6)	0.024 (6)	−0.004 (3)	0.007 (2)	0.002 (4)
C23B	0.018 (3)	0.048 (6)	0.026 (5)	−0.008 (3)	−0.002 (3)	0.003 (4)
C24B	0.016 (3)	0.063 (6)	0.026 (5)	−0.008 (4)	−0.001 (3)	0.006 (4)
C25B	0.015 (3)	0.068 (6)	0.035 (5)	0.001 (4)	0.006 (3)	0.016 (4)
C26B	0.019 (4)	0.056 (6)	0.032 (6)	0.006 (4)	0.014 (3)	0.010 (4)
C27B	0.016 (3)	0.047 (6)	0.028 (5)	−0.002 (4)	0.011 (3)	0.001 (4)
C28B	0.027 (4)	0.090 (9)	0.038 (6)	−0.015 (5)	−0.012 (4)	−0.005 (5)
C29B	0.035 (5)	0.068 (6)	0.052 (6)	0.020 (4)	0.023 (5)	0.010 (4)
C30	0.015 (2)	0.055 (4)	0.025 (3)	−0.002 (2)	0.006 (2)	0.004 (3)
C31	0.027 (3)	0.057 (4)	0.039 (3)	−0.008 (3)	0.008 (2)	−0.007 (3)
C32	0.021 (3)	0.068 (5)	0.034 (3)	−0.006 (3)	0.008 (2)	0.008 (3)

### Geometric parameters (Å, °)

**Table:**

Mo1—N1^i^	1.982 (5)	C21A—H21E	0.9600
Mo1—N3	1.995 (5)	C21A—H21F	0.9600
Mo1—N2	1.995 (5)	C20A—H20D	0.9600
Mo1—C1	2.059 (5)	C20A—H20E	0.9600
Mo1—C1^i^	2.208 (5)	C20A—H20F	0.9600
Mo1—N1	2.276 (5)	C19B—C21B	1.515 (18)
Mo1—Mo1^i^	2.5946 (8)	C19B—C20B	1.558 (19)
N1—C1	1.318 (7)	C19B—H19B	0.9800
N1—Mo1^i^	1.982 (5)	C20B—H20A	0.9600
N2—C11	1.404 (7)	C20B—H20B	0.9600
N2—C19A	1.469 (10)	C20B—H20C	0.9600
N2—C19B	1.500 (17)	C21B—H21A	0.9600
N3—C22A	1.435 (8)	C21B—H21B	0.9600
N3—C22B	1.440 (13)	C21B—H21C	0.9600
N3—C30	1.465 (8)	C22A—C23A	1.397 (10)
C1—C2	1.474 (7)	C22A—C27A	1.404 (11)
C1—Mo1^i^	2.208 (5)	C23A—C24A	1.389 (10)
C2—C3	1.408 (8)	C23A—H23A	0.9300
C2—C7	1.416 (8)	C24A—C25A	1.363 (15)
C3—C4	1.410 (7)	C24A—C28A	1.530 (13)
C3—C8	1.502 (9)	C25A—C26A	1.401 (14)
C4—C5	1.356 (10)	C25A—H25A	0.9300
C4—H4	0.9300	C26A—C27A	1.397 (10)
C5—C6	1.376 (9)	C26A—C29A	1.464 (13)
C5—C9	1.514 (8)	C27A—H27A	0.9300
C6—C7	1.399 (7)	C28A—H28A	0.9600
C6—H6	0.9300	C28A—H28B	0.9600
C7—C10	1.502 (9)	C28A—H28C	0.9600
C8—H8A	0.9600	C29A—H29A	0.9600
C8—H8B	0.9600	C29A—H29B	0.9600
C8—H8C	0.9600	C29A—H29C	0.9600
C9—H9A	0.9600	C22B—C23B	1.396 (16)
C9—H9B	0.9600	C22B—C27B	1.402 (17)
C9—H9C	0.9600	C23B—C24B	1.391 (16)
C10—H10A	0.9600	C23B—H23B	0.9300
C10—H10B	0.9600	C24B—C25B	1.36 (2)
C10—H10C	0.9600	C24B—C28B	1.527 (18)
C11—C12	1.406 (8)	C25B—C26B	1.39 (2)
C11—C16	1.408 (8)	C25B—H25B	0.9300
C12—C13	1.382 (9)	C26B—C27B	1.403 (16)
C12—H12	0.9300	C26B—C29B	1.475 (19)
C13—C14	1.387 (9)	C27B—H27B	0.9300
C13—C17	1.514 (8)	C28B—H28D	0.9600
C14—C15	1.387 (8)	C28B—H28E	0.9600
C14—H14	0.9300	C28B—H28F	0.9600
C15—C16	1.397 (8)	C29B—H29D	0.9600
C15—C18	1.501 (9)	C29B—H29E	0.9600
C16—H16	0.9300	C29B—H29F	0.9600
C17—H17A	0.9600	C30—C31	1.527 (8)
C17—H17B	0.9600	C30—C32	1.529 (7)
C17—H17C	0.9600	C30—H30	0.9800
C18—H18A	0.9600	C31—H31A	0.9600
C18—H18B	0.9600	C31—H31B	0.9600
C18—H18C	0.9600	C31—H31C	0.9600
C19A—C20A	1.507 (12)	C32—H32A	0.9600
C19A—C21A	1.547 (12)	C32—H32B	0.9600
C19A—H19A	0.9800	C32—H32C	0.9600
C21A—H21D	0.9600		
			
N1^i^—Mo1—N3	128.66 (18)	H17A—C17—H17C	109.5
N1^i^—Mo1—N2	86.9 (2)	H17B—C17—H17C	109.5
N3—Mo1—N2	104.2 (2)	C15—C18—H18A	109.5
N1^i^—Mo1—C1	101.24 (19)	C15—C18—H18B	109.5
N3—Mo1—C1	117.2 (2)	H18A—C18—H18B	109.5
N2—Mo1—C1	115.2 (2)	C15—C18—H18C	109.5
N1^i^—Mo1—C1^i^	36.16 (19)	H18A—C18—H18C	109.5
N3—Mo1—C1^i^	98.32 (19)	H18B—C18—H18C	109.5
N2—Mo1—C1^i^	115.9 (2)	N2—C19A—C20A	110.0 (9)
C1—Mo1—C1^i^	105.19 (16)	N2—C19A—C21A	116.8 (9)
N1^i^—Mo1—N1	105.27 (16)	C20A—C19A—C21A	110.1 (8)
N3—Mo1—N1	90.58 (18)	N2—C19A—H19A	106.4
N2—Mo1—N1	148.77 (18)	C20A—C19A—H19A	106.4
C1—Mo1—N1	34.94 (18)	C21A—C19A—H19A	106.4
C1^i^—Mo1—N1	88.27 (17)	N2—C19B—C21B	111.2 (18)
N1^i^—Mo1—Mo1^i^	57.82 (13)	N2—C19B—C20B	124 (2)
N3—Mo1—Mo1^i^	119.08 (14)	C21B—C19B—C20B	107.7 (16)
N2—Mo1—Mo1^i^	135.20 (15)	N2—C19B—H19B	103.9
C1—Mo1—Mo1^i^	55.23 (14)	C21B—C19B—H19B	103.9
C1^i^—Mo1—Mo1^i^	49.97 (12)	C20B—C19B—H19B	103.9
N1—Mo1—Mo1^i^	47.46 (12)	C19B—C20B—H20A	109.5
C1—N1—Mo1^i^	81.3 (3)	C19B—C20B—H20B	109.5
C1—N1—Mo1	63.5 (3)	H20A—C20B—H20B	109.5
Mo1^i^—N1—Mo1	74.73 (16)	C19B—C20B—H20C	109.5
C11—N2—C19A	120.8 (6)	H20A—C20B—H20C	109.5
C11—N2—C19B	114.1 (12)	H20B—C20B—H20C	109.5
C11—N2—Mo1	110.8 (4)	C19B—C21B—H21A	109.5
C19A—N2—Mo1	128.2 (6)	C19B—C21B—H21B	109.5
C19B—N2—Mo1	133.1 (13)	H21A—C21B—H21B	109.5
C22A—N3—C30	116.4 (6)	C19B—C21B—H21C	109.5
C22B—N3—C30	128.4 (13)	H21A—C21B—H21C	109.5
C22A—N3—Mo1	129.6 (6)	H21B—C21B—H21C	109.5
C22B—N3—Mo1	115.9 (11)	C23A—C22A—C27A	117.9 (7)
C30—N3—Mo1	113.9 (3)	C23A—C22A—N3	127.9 (8)
N1—C1—C2	129.9 (5)	C27A—C22A—N3	114.2 (7)
N1—C1—Mo1	81.6 (3)	C24A—C23A—C22A	120.3 (9)
C2—C1—Mo1	141.2 (4)	C24A—C23A—H23A	119.9
N1—C1—Mo1^i^	62.5 (3)	C22A—C23A—H23A	119.9
C2—C1—Mo1^i^	135.8 (4)	C25A—C24A—C23A	120.8 (9)
Mo1—C1—Mo1^i^	74.81 (16)	C25A—C24A—C28A	120.5 (8)
C3—C2—C7	119.7 (5)	C23A—C24A—C28A	118.7 (10)
C3—C2—C1	119.8 (5)	C24A—C25A—C26A	121.4 (8)
C7—C2—C1	120.5 (5)	C24A—C25A—H25A	119.3
C2—C3—C4	118.1 (6)	C26A—C25A—H25A	119.3
C2—C3—C8	121.9 (5)	C27A—C26A—C25A	117.3 (9)
C4—C3—C8	120.0 (6)	C27A—C26A—C29A	123.0 (10)
C5—C4—C3	122.7 (6)	C25A—C26A—C29A	119.7 (8)
C5—C4—H4	118.7	C26A—C27A—C22A	122.3 (8)
C3—C4—H4	118.7	C26A—C27A—H27A	118.8
C4—C5—C6	118.7 (5)	C22A—C27A—H27A	118.8
C4—C5—C9	121.3 (7)	C23B—C22B—C27B	119.5 (13)
C6—C5—C9	120.0 (7)	C23B—C22B—N3	115.1 (14)
C5—C6—C7	122.3 (6)	C27B—C22B—N3	125.2 (14)
C5—C6—H6	118.9	C24B—C23B—C22B	120.6 (16)
C7—C6—H6	118.9	C24B—C23B—H23B	119.7
C6—C7—C2	118.3 (6)	C22B—C23B—H23B	119.7
C6—C7—C10	118.3 (6)	C25B—C24B—C23B	118.2 (16)
C2—C7—C10	123.4 (5)	C25B—C24B—C28B	121.6 (16)
C3—C8—H8A	109.5	C23B—C24B—C28B	120.1 (18)
C3—C8—H8B	109.5	C24B—C25B—C26B	124.2 (15)
H8A—C8—H8B	109.5	C24B—C25B—H25B	117.9
C3—C8—H8C	109.5	C26B—C25B—H25B	117.9
H8A—C8—H8C	109.5	C25B—C26B—C27B	116.7 (16)
H8B—C8—H8C	109.5	C25B—C26B—C29B	122.8 (15)
C5—C9—H9A	109.5	C27B—C26B—C29B	120.4 (17)
C5—C9—H9B	109.5	C22B—C27B—C26B	120.7 (15)
H9A—C9—H9B	109.5	C22B—C27B—H27B	119.6
C5—C9—H9C	109.5	C26B—C27B—H27B	119.6
H9A—C9—H9C	109.5	C24B—C28B—H28D	109.5
H9B—C9—H9C	109.5	C24B—C28B—H28E	109.5
C7—C10—H10A	109.5	H28D—C28B—H28E	109.5
C7—C10—H10B	109.5	C24B—C28B—H28F	109.5
H10A—C10—H10B	109.5	H28D—C28B—H28F	109.5
C7—C10—H10C	109.5	H28E—C28B—H28F	109.5
H10A—C10—H10C	109.5	C26B—C29B—H29D	109.5
H10B—C10—H10C	109.5	C26B—C29B—H29E	109.5
N2—C11—C12	125.1 (6)	H29D—C29B—H29E	109.5
N2—C11—C16	118.4 (5)	C26B—C29B—H29F	109.5
C12—C11—C16	116.5 (5)	H29D—C29B—H29F	109.5
C13—C12—C11	121.6 (6)	H29E—C29B—H29F	109.5
C13—C12—H12	119.2	N3—C30—C31	116.4 (5)
C11—C12—H12	119.2	N3—C30—C32	111.2 (5)
C12—C13—C14	119.7 (5)	C31—C30—C32	111.4 (5)
C12—C13—C17	120.7 (7)	N3—C30—H30	105.7
C14—C13—C17	119.5 (6)	C31—C30—H30	105.7
C13—C14—C15	121.4 (6)	C32—C30—H30	105.7
C13—C14—H14	119.3	C30—C31—H31A	109.5
C15—C14—H14	119.3	C30—C31—H31B	109.5
C14—C15—C16	117.7 (6)	H31A—C31—H31B	109.5
C14—C15—C18	122.3 (6)	C30—C31—H31C	109.5
C16—C15—C18	120.0 (5)	H31A—C31—H31C	109.5
C15—C16—C11	122.9 (5)	H31B—C31—H31C	109.5
C15—C16—H16	118.5	C30—C32—H32A	109.5
C11—C16—H16	118.5	C30—C32—H32B	109.5
C13—C17—H17A	109.5	H32A—C32—H32B	109.5
C13—C17—H17B	109.5	C30—C32—H32C	109.5
H17A—C17—H17B	109.5	H32A—C32—H32C	109.5
C13—C17—H17C	109.5	H32B—C32—H32C	109.5
			
N1^i^—Mo1—N1—C1	87.8 (3)	C4—C5—C6—C7	3.0 (9)
N3—Mo1—N1—C1	−141.6 (3)	C9—C5—C6—C7	−176.3 (6)
N2—Mo1—N1—C1	−22.3 (5)	C5—C6—C7—C2	0.3 (8)
C1^i^—Mo1—N1—C1	120.1 (3)	C5—C6—C7—C10	−177.1 (6)
Mo1^i^—Mo1—N1—C1	87.8 (3)	C3—C2—C7—C6	−4.2 (8)
N1^i^—Mo1—N1—Mo1^i^	0.0	C1—C2—C7—C6	175.6 (5)
N3—Mo1—N1—Mo1^i^	130.57 (16)	C3—C2—C7—C10	173.0 (5)
N2—Mo1—N1—Mo1^i^	−110.1 (3)	C1—C2—C7—C10	−7.3 (8)
C1—Mo1—N1—Mo1^i^	−87.8 (3)	C19A—N2—C11—C12	44.6 (9)
C1^i^—Mo1—N1—Mo1^i^	32.26 (16)	C19B—N2—C11—C12	64.3 (12)
N1^i^—Mo1—N2—C11	−178.4 (4)	Mo1—N2—C11—C12	−129.7 (6)
N3—Mo1—N2—C11	52.4 (4)	C19A—N2—C11—C16	−138.3 (7)
C1—Mo1—N2—C11	−77.4 (4)	C19B—N2—C11—C16	−118.6 (11)
C1^i^—Mo1—N2—C11	159.2 (4)	Mo1—N2—C11—C16	47.4 (6)
N1—Mo1—N2—C11	−63.5 (5)	N2—C11—C12—C13	176.9 (6)
Mo1^i^—Mo1—N2—C11	−142.5 (3)	C16—C11—C12—C13	−0.3 (9)
N1^i^—Mo1—N2—C19A	7.8 (6)	C11—C12—C13—C14	−2.5 (10)
N3—Mo1—N2—C19A	−121.3 (6)	C11—C12—C13—C17	177.8 (6)
C1—Mo1—N2—C19A	108.8 (6)	C12—C13—C14—C15	2.6 (9)
C1^i^—Mo1—N2—C19A	−14.6 (7)	C17—C13—C14—C15	−177.8 (6)
N1—Mo1—N2—C19A	122.7 (7)	C13—C14—C15—C16	0.2 (9)
Mo1^i^—Mo1—N2—C19A	43.7 (7)	C13—C14—C15—C18	178.4 (6)
N1^i^—Mo1—N2—C19B	−15.9 (11)	C14—C15—C16—C11	−3.2 (9)
N3—Mo1—N2—C19B	−145.1 (11)	C18—C15—C16—C11	178.5 (6)
C1—Mo1—N2—C19B	85.1 (12)	N2—C11—C16—C15	−174.1 (5)
C1^i^—Mo1—N2—C19B	−38.3 (12)	C12—C11—C16—C15	3.3 (9)
N1—Mo1—N2—C19B	99.0 (12)	C11—N2—C19A—C20A	38.5 (11)
Mo1^i^—Mo1—N2—C19B	19.9 (12)	C19B—N2—C19A—C20A	−35 (5)
N1^i^—Mo1—N3—C22A	−14.6 (8)	Mo1—N2—C19A—C20A	−148.3 (6)
N2—Mo1—N3—C22A	82.9 (7)	C11—N2—C19A—C21A	−88.0 (10)
C1—Mo1—N3—C22A	−148.4 (7)	C19B—N2—C19A—C21A	−162 (6)
C1^i^—Mo1—N3—C22A	−36.5 (7)	Mo1—N2—C19A—C21A	85.2 (11)
N1—Mo1—N3—C22A	−124.9 (7)	C11—N2—C19B—C21B	−100.4 (19)
Mo1^i^—Mo1—N3—C22A	−85.0 (7)	C19A—N2—C19B—C21B	15 (4)
N1^i^—Mo1—N3—C22B	−31.6 (15)	Mo1—N2—C19B—C21B	98 (2)
N2—Mo1—N3—C22B	66.0 (15)	C11—N2—C19B—C20B	31 (3)
C1—Mo1—N3—C22B	−165.3 (15)	C19A—N2—C19B—C20B	146 (7)
C1^i^—Mo1—N3—C22B	−53.4 (15)	Mo1—N2—C19B—C20B	−131 (2)
N1—Mo1—N3—C22B	−141.8 (15)	C22B—N3—C22A—C23A	−172 (7)
Mo1^i^—Mo1—N3—C22B	−102.0 (15)	C30—N3—C22A—C23A	−40.2 (16)
N1^i^—Mo1—N3—C30	162.3 (3)	Mo1—N3—C22A—C23A	136.7 (11)
N2—Mo1—N3—C30	−100.1 (4)	C22B—N3—C22A—C27A	10 (5)
C1—Mo1—N3—C30	28.5 (4)	C30—N3—C22A—C27A	141.9 (9)
C1^i^—Mo1—N3—C30	140.4 (4)	Mo1—N3—C22A—C27A	−41.2 (14)
N1—Mo1—N3—C30	52.1 (4)	C27A—C22A—C23A—C24A	0.8 (18)
Mo1^i^—Mo1—N3—C30	91.9 (3)	N3—C22A—C23A—C24A	−177.0 (11)
Mo1^i^—N1—C1—C2	−128.2 (5)	C22A—C23A—C24A—C25A	−0.4 (16)
Mo1—N1—C1—C2	154.6 (6)	C22A—C23A—C24A—C28A	178.3 (11)
Mo1^i^—N1—C1—Mo1	77.19 (15)	C23A—C24A—C25A—C26A	0.3 (14)
Mo1—N1—C1—Mo1^i^	−77.19 (15)	C28A—C24A—C25A—C26A	−178.3 (9)
N1^i^—Mo1—C1—N1	−100.6 (3)	C24A—C25A—C26A—C27A	−0.7 (14)
N3—Mo1—C1—N1	44.3 (4)	C24A—C25A—C26A—C29A	−179.6 (9)
N2—Mo1—C1—N1	167.4 (3)	C25A—C26A—C27A—C22A	1.1 (15)
C1^i^—Mo1—C1—N1	−63.7 (3)	C29A—C26A—C27A—C22A	180.0 (11)
Mo1^i^—Mo1—C1—N1	−63.7 (3)	C23A—C22A—C27A—C26A	−1.2 (18)
N1^i^—Mo1—C1—C2	111.1 (7)	N3—C22A—C27A—C26A	176.9 (9)
N3—Mo1—C1—C2	−104.0 (7)	C22A—N3—C22B—C23B	10 (4)
N2—Mo1—C1—C2	19.1 (7)	C30—N3—C22B—C23B	−48 (3)
C1^i^—Mo1—C1—C2	148.0 (8)	Mo1—N3—C22B—C23B	148 (2)
N1—Mo1—C1—C2	−148.3 (9)	C22A—N3—C22B—C27B	−165 (9)
Mo1^i^—Mo1—C1—C2	148.0 (8)	C30—N3—C22B—C27B	136 (3)
N1^i^—Mo1—C1—Mo1^i^	−36.96 (18)	Mo1—N3—C22B—C27B	−27 (4)
N3—Mo1—C1—Mo1^i^	107.96 (19)	C27B—C22B—C23B—C24B	3(5)
N2—Mo1—C1—Mo1^i^	−128.88 (19)	N3—C22B—C23B—C24B	−173 (2)
C1^i^—Mo1—C1—Mo1^i^	0.0	C22B—C23B—C24B—C25B	0(4)
N1—Mo1—C1—Mo1^i^	63.7 (3)	C22B—C23B—C24B—C28B	178 (3)
N1—C1—C2—C3	165.0 (5)	C23B—C24B—C25B—C26B	−2(4)
Mo1—C1—C2—C3	−57.7 (8)	C28B—C24B—C25B—C26B	179 (2)
Mo1^i^—C1—C2—C3	75.2 (7)	C24B—C25B—C26B—C27B	2(4)
N1—C1—C2—C7	−14.8 (8)	C24B—C25B—C26B—C29B	−178 (2)
Mo1—C1—C2—C7	122.6 (6)	C23B—C22B—C27B—C26B	−3(5)
Mo1^i^—C1—C2—C7	−104.6 (6)	N3—C22B—C27B—C26B	173 (3)
C7—C2—C3—C4	4.8 (8)	C25B—C26B—C27B—C22B	1(4)
C1—C2—C3—C4	−174.9 (5)	C29B—C26B—C27B—C22B	−179 (3)
C7—C2—C3—C8	−173.3 (5)	C22A—N3—C30—C31	72.3 (8)
C1—C2—C3—C8	7.0 (8)	C22B—N3—C30—C31	90.8 (15)
C2—C3—C4—C5	−1.6 (9)	Mo1—N3—C30—C31	−105.1 (4)
C8—C3—C4—C5	176.5 (6)	C22A—N3—C30—C32	−56.7 (8)
C3—C4—C5—C6	−2.3 (9)	C22B—N3—C30—C32	−38.2 (16)
C3—C4—C5—C9	176.9 (6)	Mo1—N3—C30—C32	125.9 (4)

Symmetry codes: (i) −*x*+2, −*y*+2, −*z*+1.
